# Establishment of long-term serum-free culture for lacrimal gland stem cells aiming at lacrimal gland repair

**DOI:** 10.1186/s13287-019-1541-1

**Published:** 2020-01-08

**Authors:** Sa Xiao, Yan Zhang

**Affiliations:** MOE Key Laboratory of Gene Function and Regulation, School of Life Sciences, Sun Yat-sen University, Guangzhou, 510006 Guangdong People’s Republic of China

**Keywords:** Lacrimal gland stem cell, Dry eye disease, 3D culture, Serum-free, Stem cell therapy

## Abstract

**Background:**

Aqueous-deficient dry eye disease (ADDED) resulting from dysfunction of the lacrimal gland (LG) is currently incurable. Although LG stem/progenitor cell-based therapy is considered to be a promising strategy for ADDED patients, the lack of a reliable serum-free culture method to obtain enough lacrimal gland stem cells (LGSCs) and the basic standard of LGSC transplantation are obstacles for further research.

**Methods:**

Adult mouse LGSCs were cultured in Matrigel-based 3D culture under serum-free culture condition, which contained EGF, FGF10, Wnt3A, and Y-27632. LGSCs were continuously passaged over 40 times every 7 days, and the morphology and cell numbers were recorded. LGSCs were induced to differentiate to ductal cells by reducing Matrigel rigidity, while fetal bovine serum was used for the induction of acinar cells. RT-PCR or qRT-PCR analysis, RNA-sequence analysis, H&E staining, and immunofluorescence were used for characterization and examining the differentiation of LGSCs. LGSCs were allotransplanted into diseased LGs to examine the ability of repairing the damage. The condition of eye orbits was recorded using a camera, the tear production was measured using phenol red-impregnated cotton threads, and the engraftments of LGSCs were examined by immunohistochemistry.

**Results:**

We established an efficient 3D serum-free culture for adult mouse LGSCs, in which LGSCs could be continuously passaged for long-term expansion. LGSCs cultured from both the healthy and ADDED mouse LGs expressed stem/progenitor cell markers Krt14, Krt5, P63, and nestin, had the potential to differentiate into acinar or ductal-like cells in vitro and could engraft into diseased LGs and relieve symptoms of ADDED after orthotopic injection of LGSCs.

**Conclusion:**

We successfully established an efficient serum-free culture for adult mouse LGSCs aiming at LG repair for the first time. Our approach provides an excellent theoretical and technical reference for future clinical research for ADDED stem cell therapy.

## Background

The lacrimal gland (LG) plays an indispensable role in maintaining a homeostatic microenvironment for the ocular surface. It can produce the aqueous layer component of the tear film to lubricate the ocular surface and protect the epithelia of the cornea and conjunctiva from environmental harm. Aqueous-deficient dry eye disease (ADDED) is caused by the lack of tear secretion resulting from dysfunction or degeneration of the lacrimal gland, such as Sjogren’s syndrome (SjS). ADDED can lead to severe symptoms, including inflammation of the eyes, damage to the corneal epithelia, and loss of vision, which affect the quality of daily life of millions of people.

Currently, the prevalence of dry eye disease (DED) ranges from about 5 to 30% in various age populations [1], and about 50% of DED patients have aqueous deficiency [2]. The treatment options for DED include artificial tears or drug stimulation of tear secretion, which can achieve lubrication of the dry ocular surface. Nevertheless, all these treatments cannot cure DED and only provide temporary relief of the symptoms. Hence, there is an urgent need for developing a new treatment to cure ADDED to improve the life quality of the patients.

Stem/progenitor cell-based therapies are considered alternative approaches for treating incurable diseases [3]. In exocrine glands, stem cells have been shown to be viable for regenerating or repairing damage, such as in the pancreas [4], salivary gland [5, 6], and mammary gland [7]. LG stem cells are the source of acinar and ductal cells, which could be used to repair the severe inflammatory damage induced by interleukin-1α (IL-1α) [8].

Recently, some researchers have demonstrated that c-kit^+^dim/EpCAM^+^/Sca1^−^/CD34^−^/CD45^−^ cells directly sorted from adult mouse LGs (efficiency of 2.2–2.7%) via fluorescence-activated cell sorting (FACS) have the characteristic of LG progenitor cells, named epithelial cell progenitors (EPCPs). EPCPs have shown engraftment ability and have improved the structure and function of a diseased LG after injection into the LG of *TSP-1*^*−/−*^ mice with human Sjogren’s syndrome [9]. Due to the low efficiency of FACS, a massive number of LG cells are needed to sort out EPCPs. In addition, there are few reports on serum-free culture for LG cells aiming at clinical use. Therefore, obtaining enough cells for therapeutic application is an enormous challenge, and developing a new strategy with high efficiency for LG stem/progenitor cell isolation and culture is needed.

In this study, we established an adult lacrimal gland stem cell (LGSC) culture via optimizing the serum-free culture medium and using a 3D culture strategy. The LGSCs directly cultured from both healthy and ADDED LGs showed the robust capacity of self-renewal and proliferation, engraftment into the ADDED mouse LGs, and improvement of tear production. Our work provides a promising pathway for the allograft and autograft of LGSCs from patients in ADDED therapy studies.

## Methods

### Mice

C57BL/6 (6–8-week-old) mice from the Model Animal Research Center of Sun Yat-sen University were used for the LGSC culture and characterization. ROSA26^mT/mG^ mice and NOD/ShiLtJ mice were purchased from the Model Animal Research Center of Nanjing University and were bred in the Model Animal Research Center of Sun Yat-sen University. The ROSA-LGSC donor cells were obtained from ROSA26^mT/mG^ mice. NOD/ShiLtJ mice were the recipients and were used for the NOD-LGSC culture.

### LGSC primary culture and maintenance

For the LGSC primary culture, 6–8-week-old mice were sacrificed. Then the LGs were cut into small fragments (about 1 mm^3^), treated with 25 U/ml Dispase (BD Biosciences) and 0.1% Collagenase I (Gibco) for 1 h at 37 °C. They were then treated with 0.05% trypsin (Sigma) for 10 min at 37 °C to dissociate into single cells by pipetting. A total of 1 × 10^4^ cells were seeded into 80 μl of Matrigel-Lacrimal gland stem cell medium (LGSCM) matrix (Matrigel: LGSCM = 1:1) in each well of a 24-well plate. The well was pre-coated with 20 μl Matrigel-LGSCM matrix. After incubation for 20 min at 37 °C, the mix was solidified and then 600 μl LGSCM was added, which contained DMEM/F12 (1:1 mixture of Dulbecco’s modified Eagle’s medium and Ham’s F-12) (Sigma), 1× N2 (Gibco), 1× B27 (Gibco), 2 mM L-glutaMAX (Gibco), 0.1 mM NEAA (non-essential amino acids, Gibco), 50 ng/ml murine epidermal growth factor (EGF) (PeproTech), 100 ng/ml fibroblast growth factor (FGF)10 (PeproTech), Wnt3A 10 ng/ml (PeproTech), and 10 μM Y-27632 (Selleck).

For LGSC maintenance and passage, LGSC spheres cultured for 7 days were released by incubation in 10 U/ml Dispase for 1 h at 37 °C. They were then treated with 0.05% trypsin for 5 min at 37 °C to dissociate single cells, and the single cells were planted as in the method for a primary culture.

### Measurement of LGSC spheres

To measure the diameter of LGSC spheres in different conditions, five fields of LGSC spheres under a microscope were obtained randomly in each condition. Then the diameters of all the spheres were measured with NIS-Element software (Nikon).

### LGSC differentiation

For LGSC differentiation, three procedures were performed. First, the culture time of LGSCs was elongated from 7 to 14 days without changing the medium or the rigidity of the Matrigel-LGSCM matrix (1:1) for the random differentiation. Then, the ratio of the Matrigel-LGSCM matrix was reduced from 1:1 to 1:2 for the differentiation of ductal cells. When the LGSCs were seeded into the matrix, the induction was continued for 14 days. Finally, 10% FBS (Hyclone) was added into the LGSCM for the differentiation of acinar cells from the beginning of the passage, and the induction also lasted 14 days.

### LGSC transplantation

The NOD/ShiLtJ mice that were more than 12 weeks old were appropriate for LGSC orthotopic injection. The LGSC spheres cultured for 7 or 14 days and passaged at least five times were digested into single cells with 0.05% trypsin for 5 min at 37 °C. They were then resuspended with DMEM/F12 at a density of 2 × 10^6^ cells/ml. After being anesthetized, approximately 2 × 10^4^ cells were injected into the LG of the recipients by glass capillary with an internal diameter of 100–150 μm, and the vehicle (DMEM/F12) was injected into the contralateral LGs as control. The analysis of LGSC engraftment was performed at 8 weeks after injection.

### Quantification of LGSC engraftment and ratio of lymphatic infiltration

The Image-Pro Plus software was used for quantification of LGSC engraftment and ratio of lymphatic infiltration. The positive areas of LGSC engraftment were selected and divided by the total cell areas, and the result was the engraftment efficiency. The ratio of lymphatic infiltration was quantified by the same method.

### Measurement of tear secretion volume

The tear secretion volume was measured with home-made phenol red-impregnated cotton threads, which were similar to the threads described in a previous study [8, 9]. The threads were put at the canthus without touching the ocular surface of the anesthetized mice for 10 s. The length of the thread, which turned red after wetting with tears, was measured as the tear volume.

### Flow cytometry

The LGSC spheres were digested into single cells with 0.05% trypsin for 5 min at 37 °C and were then fixed in 4%PFA for 10 min. The single cells were treated with blocking buffer (PBS, 10% normal non-immune goat serum and 0.01% Triton) for 30 min and were then incubated with diluted primary antibodies Anti-Krt14 (conjugated with FITC, 1:10, Abcam) for 30 min at 4 °C. Finally, the stained cells were measured with a FACS LSR-II Flow Cytometer (BD). The control was an isotype.

### Immunostaining

The tissues and LGSC spheres were fixed in 4% paraformaldehyde (PFA) for 24 h. Both paraffin and frozen sections were prepared for immunofluorescence (IF), while only paraffin sections were used for immunohistochemistry (IHC). Before incubating the primary antibodies, paraffin sections were dewaxed and boiled in 10 mM citric acid retrieval buffer (pH 6.0) for 17 min. For IF staining, the primary antibodies (Additional file 1: Table S1) were diluted in blocking buffer (PBS, 10% normal non-immune goat serum and 0.01% Triton) and incubated at room temperature for 2 h. Then the sections were treated with the secondary antibodies for 1 h at room temperature in the darkroom. The secondary antibodies are provided in Additional file 1: Table S2. Nuclear staining was performed with DAPI (Sigma-Aldrich) for 5 min. For IHC staining, the sections were treated with secondary antibodies and conjugated with horseradish peroxidase (HRP) and 3,3′-diaminobenzidine (Maxin Biotechnologies). Nuclear staining was performed with hematoxylin.

### Hematoxylin and eosin staining

The paraffin sections were dewaxed and washed in PBS and then were treated with hematoxylin for 5 min and washed in flow water for 10 min. They were then stained with eosin for 1 min and dehydrated in the ethanol and xylene.

### RT-PCR and qRT-PCR

Total RNA was extracted using Trizol reagent (Invitrogen). We then performed reverse transcription using a ReverTra Ace® qPCR RT Master Mix (Toyobo) for first-strand cDNA. PCR was performed using rTaq (Takara) with the reaction conditions according to the manufacturer’s recommendations: initial hold at 94 °C for 5 min and then 35 cycles at 94 °C for 30 s, 60 °C for 30 s, and 72 °C for 60 s. In addition, the qRT-PCR was performed using a LightCycler 480 SYBR Green Master I Mix (Roche). According to the manufacturer’s recommendations, the program was as follows: initial hold at 95 °C for 5 min and then 40 cycles at 95 °C for 10 s, 60 °C for 20 s, and 72 °C for 20 s. All the primers are listed in Additional file 2: Table S3.

### RNA sequence

Total RNA of LGSCs, embryonic LGs, and LGs were extracted using Trizol reagent (Invitrogen). cDNA libraries were generated using NEBNext® UltraTM RNA Library Prep Kit for Illumina® (NEB, USA) and the quality of the cDNA libraries were assessed on the Agilent Bioanalyzer 2100 system. The RNA sequence was performed on an Illumina platform by Novogene (Beijing, China) and 150-bp paired-end reads were generated. CASAVA was used for basecalls and featuerCounts (1.5.0-p3) was used for estimating the abundances. The differential expression analyses were performed by DESeq2 R package (1.16.1). Gene Ontology (GO) and KEGG enrichment analyses were implemented by the clusterProfiler R package. The data of differential gene expression analysis between LGSCs day 7 and day 14 are shown in Additional file 3: Table S4.

### Image analysis

Immunofluorescence images from LGSC-sphere sections were acquired with a Nikon microscope (Nikon). Immunohistochemistry images from tissue and LGSC-sphere sections and images of the LGSCs were acquired with an Olympus microscope (Olympus).

### Statistical analysis

All values were presented as the mean ± SEM. The statistical analysis was performed using GraphPad Prism 5 software. An unpaired two-tailed Student’s *t* test was used to determine *P* values for statistical significance.

## Results

### Isolation and characterization of LGSCs

**Fig. 1:**
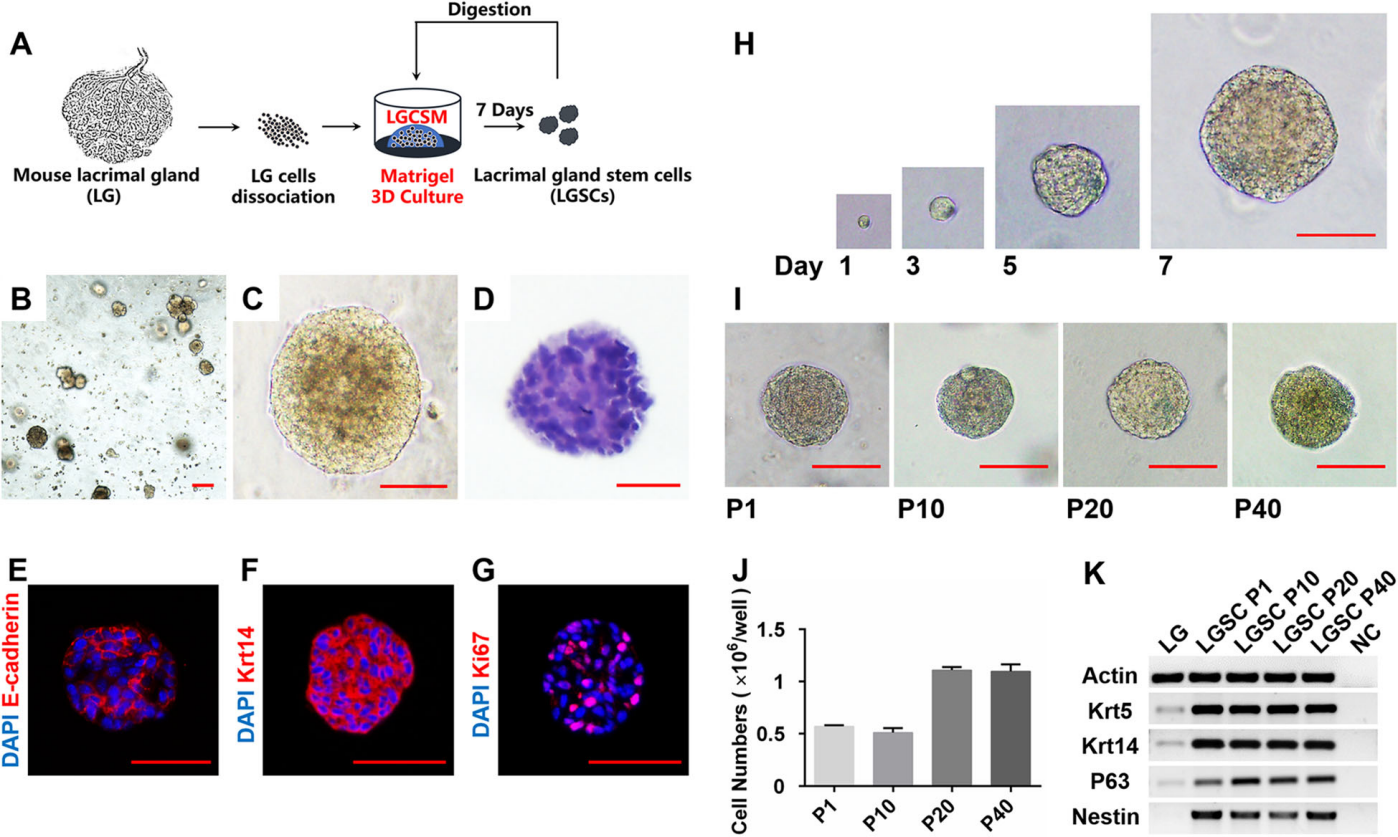
Isolation and characterization of lacrimal gland stem cells (LGSGs). **a** The strategy of LGSG primary and continuous passage culture. **b**, **c** The morphology of LGSCs in primary culture at day 7; scale bar, **b** 200 μm, **c** 100 μm. **d** Hematoxylin and eosin staining of LGSCs at day 7; scale bar, 50 μm. **e–g** Immunofluorescent staining of LGSCs at day 7. **e** LGSCs express epithelial cell marker E-cadherin (red), scale bar, 50 μm; **f** LGSCs express stem cell marker Krt14 (red), scale bar, 50 μm; **g** LGSCs express proliferative cell marker Ki67 (red), scale bar, 50 μm. Counterstain, DAPI (4′,6-diamidino-2-phenylindole, blue). **h, i** The morphology of LGSCs in continuous passage culture. **h** LGSGs cultured for 1, 3, 5, and 7 days, scale bar, 100 μm; **i** LGSCs cultured in different passages (P1, P10, P20, and P40), scale bar, 100 μm. **j** The cell numbers of LGSCs cultured for 7 days in different passages (P1, P10, P20, and P40). The cells were seeded at 1 × 10^4^ cells per well (24-well plate). The experiments were performed three times and the average cell number is shown in the graph. **k** Transcriptional expression of adult stem/progenitor cell markers of the mouse LG and different passage LGSCs (P1, P10, P20, and P40). LG, lacrimal gland; NC, negative control

To obtain adult mouse LGSCs, we established a Matrigel-based 3D serum-free culture supplemented with LGSCM (Fig. 1a) that we optimized. After the 7-day primary culture, the single cells formed spheres with most having a > 100 μm diameter (Fig. 1b, c). The LGSCM mainly contained epidermal growth factor (EGF), fibroblast growth factor (FGF)10, canonical Wnt signaling protein (Wnt3A), and the Rho-associated protein kinase (ROCK) inhibitor, Y-27632. In the primary culture, either cell numbers or diameters of the spheres were significantly decreased in the absence of EGF, Y-27632, or FGF10, respectively (Additional file 4: Figure S1 A–C), whereas there was no obvious change in the absence of Wnt3A. However, in the passage culture, a significant reduction of cell numbers or diameters of the spheres was shown in the absence of Wnt3A, compared to the LGSCM condition (Additional file 4: Figure S1 D–F), and the spheres that formed in the condition of withdrawing EGF, Y-27632, or FGF10 could not be subcultured (data not shown). These results suggested that EGF, FGF10, and Y-27632 were essential for the culture of LGSCs, and Wnt3A was also necessary for the passage culture and cell sustainable growth.

Hematoxylin and eosin (H&E) staining and IF analysis showed that the spheres obtained from the 3D serum-free culture were solid (Fig. 1d) and all of the spheroid cells expressed the epithelial cell markers [9, 10], E-cadherin (Fig. 1e) and Epcam (Additional file 5: Figure S2 A), while endothelial cell marker VEGFR2 or fibroblast marker FAP-α could not be detected (Additional file 5: Figure S2 A), suggesting that the spheroid cells were epithelia rather than endothelial cells or fibroblasts. Previous studies indicated that Krt14 is the marker for stem/progenitor cells of secretory glands [11–13]. In the adult mouse LG, the basal cells of ducts and myoepithelial cells express keratin (Krt)14, suggesting that these cells might have the characteristics of lacrimal gland stem cells (LGSCs; [14]. In the present study, we found that all the spheroid cells highly expressed Krt14 (Fig. 1f), while over half of the spheroid cells expressed Ki67 (Fig. 1g), a marker for proliferation [15]. These data suggested the cells we obtained were the stem cells of the adult LG with robust self-renewal and proliferation capacity. Hence, we presumed that the spheres were composed of LG stem cells, named LGSC spheres.

As the long-term in vitro expansion of LGSCs has been an unsolved problem, we attempted to continuously passage LGSCs under serum-free culture condition. When the diameter of the spheres reached approximately 100 μm, the spheroid cells were passaged. LGSC spheres were digested enzymatically into single cells and seeded into 24-well plates at a density of 1 × 10^4^ cells/well every 7 days (Fig. 1a, h). Our results showed that LGSC spheres could be passaged at least 40 times and they maintained morphology as well as a high growth rate (Fig. 1i, j).

Keratin (Krt)5 could form heterodimers with Krt14, also known as a stem cell marker [16–18]. In addition, nestin and P63 are also used as gland stem/progenitor cell markers [17, 19, 20]. In this study, we detected stem cell markers in the LGSC spheres in different passage numbers and found that LGSC spheres in different passages constantly highly expressed Krt14 and Krt5 (Fig. 1k). In addition, nestin and P63 were also detected in LGSC spheres (Fig. 1k). These results indicated that LGSC spheres maintained the stem cell characteristics during the long-term in vitro expansion and gave further support to our hypothesis that LGSC spheres were composed of LGSCs.

### LGSCs differentiate into acinar and ductal cells in vitro

Matrigel-based 3D culture has been reported to cultivate organoids from different epithelial tissues, such as the pancreas [21], mammary gland [22], and salivary gland [23, 24]. These organoids were tissue/organ-specific mini-organs containing stem cells and cells in differentiation stages. An appropriate 3D culture condition could provide the desired microenvironment for maintaining stem cell characteristics or promoting differentiation [25].

**Fig. 2:**
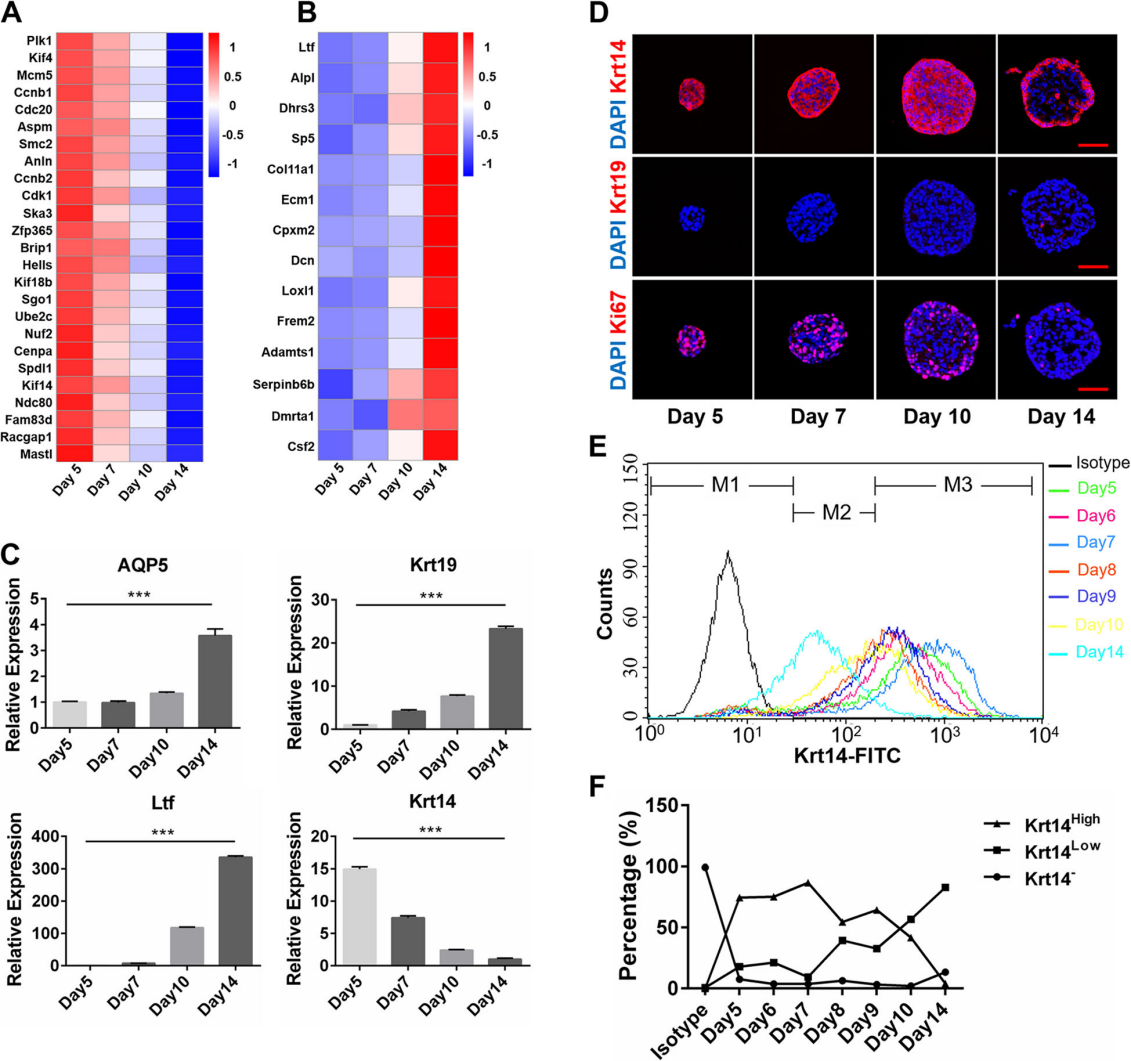
Differentiation potential of LGSCs in vitro. **a, b** Heatmap of RNA-sequence analysis of LGSCs cultured for 5, 7, 10, and 14 days. **a** Heatmap of cell division genes; **b** heatmap of cell differentiation and gland development; *n* = 3. **c** qRT-PCR analysis of adult stem cell and differentiated markers of LGSCs cultured for 5, 7, 10, and 14 days. Secretory cell marker AQP5, secretory protein gene of LG Ltf, and ductal cell marker Krt19 are significantly upregulated. Adult stem cell marker Krt14 is significantly downregulated, ***, *P* < 0.01; *n* = 3. **d** Immunofluorescent staining of LGCSs cultured for 5, 7, 10, and 14 days. As elongating culture time, cells expressing Krt14 (red) and Ki67 (red) are significantly decreased, while cells expressing Krt19 (red) emerge, scale bar, 50 μm. Nuclear staining, DAPI (blue). **e, f** Flow cytometer analysis of Krt14 expression of LGSCs cultured for 5, 6, 7, 8, 9, 10, and 14 days. **e** Flow cytometric profile, M1 (Krt14^−^), M2 (Krt14^Low^), M3 (Krt14^High^); **f** comparative graph of LGSC subpopulations shows that LGSCs cultured for 7 days have the highest proportion of Krt14-positive cells

For analyzing the dynamic transcription alteration during 3D culture, LGSC spheres cultured for different times were collected for transcriptional analysis. The heatmaps of RNA-sequence analysis showed that gland development and differentiation-related genes were upregulated as elongating culture times, while cell division genes were downregulated (Fig. 2a, b). The data from qRT-PCR also confirmed these results. Expressions of acinar marker aquaporin (AQP)5, ductal marker keratin (Krt)19, and lacrimal secretory protein lactotransferrin (Ltf) [26], were significantly upregulated in LGSC spheres cultured for 14 days compared to LGSC spheres cultured for 5 and 7 days, while expression of stem cell maker Krt14 was significantly downregulated in elongating culture times (Fig. 2c). Furthermore, IF analysis demonstrated that the cells expressing Krt14 and Ki67 were significantly decreased and mostly located in the outer layers of LGSC spheres, while a few cells expressing Krt19 arose from the inner layers of LGSC spheres (Fig. 2d). Hence, these results suggested that the proliferation of LGSC spheres gradually ceased when LGSC spheres differentiated into acinar or ductal cells.

We then analyzed the percentage of Krt14^+^ cells in LGSC spheres during differentiation by flow cytometry (FCM) and revealed three populations: Krt14^−^, Krt14^Low^, and Krt14^High^. The ratio of the Krt14^High^ population reached a peak in 7-day cultures and dramatically decreased in 14-day cultures (Fig. 2e, f). Furthermore, the ability of LGSC sphere formation of 7 day-cultured LGSCs and 14 day-cultured ones were detected. LGSCs cultured for 7 days showed significantly higher ability of sphere formation than LGSCs cultured for 14 days (Additional file 5: Figure S2 B–D). These results suggested that LGSC spheres cultured for 7 days might have the highest proportion of stem cells and self-renewal ability.

**Fig. 3:**
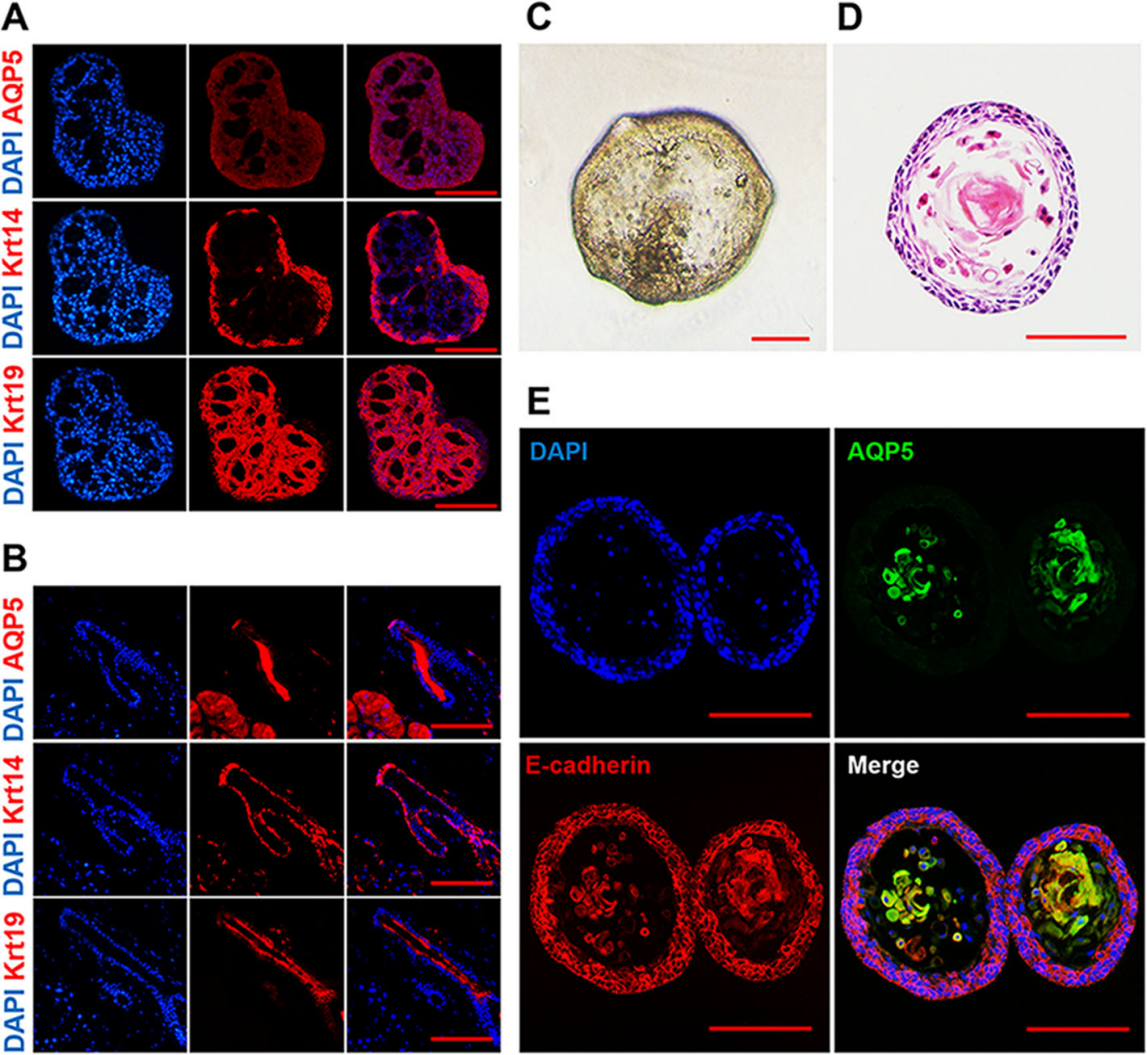
LGSC differentiation into acinar and ductal-like cells in vitro. **a** Immunofluorescent staining of LGCSs cultured for 14 days in 1/3 Matrigel. Most of the inner layer cells express Krt19 (red) and the outer layer cells express Krt14 (red); all cells express AQP5 (red); scale bar, 100 μm. Nuclear staining, DAPI (blue). **b** Immunofluorescent staining of mouse LG. Suprabasal cells located in inner layers of duct express Krt19 (red) and basal cells located in outer layers of duct express Krt14 (red); all secretory cells highly express AQP5 (red); scale bar, 100 μm. Nuclear staining, DAPI (blue). **c** The morphology of the LGSC culture for 14 days with addition of FBS; scale bar, 100 μm. **d** H&E staining of the LGSC culture for 14 days with addition of FBS. Acinar-like cells emerge in the LGSC sphere; scale bar,100 μm. **e** Immunofluorescent staining of the LGSC culture for 14 days with addition of FBS. The acinar-like cells highly express AQP5 (green), and all cells express epithelial cell marker E-cadherin (red); scale bar, 100 μm. Nuclear staining, DAPI (blue)

Development and morphogenesis of the LG are regulated by epithelium-mesenchyme interactions, the degradation of the extracellular matrix (ECM) releasing the signals for cell elongation and providing the spatiotemporal domain of cell proliferation. The degradation of the ECM could reduce the rigidity of the microenvironment for branching morphogenesis and ductal formation in the embryonic LG [27]. Thus, we reduced the Matrigel concentration to one third of the original to reduce the rigidity of the matrix for mimicking the ECM degradation to induce ductal differentiation of LGSCs. After 14-day culture in the 1/3 Matrigel condition, there were cavities emerging inside the LGSC spheres, of which most of the inner layer cells expressed Krt19 and the outer layer cells expressed Krt14 (Fig. 3a), similar to the LG ductal structure (Fig. 3b), suggesting that LGSCs could differentiate into LG duct-like cells.

The mature acinar cells are larger in size and have much lower nucleo-cytoplasmic ratios than immature acinar cells [28]. Although AQP5 was detected in LGSC spheres after induction in the one third Matrigel condition, the morphology of the cells was still similar to LGSCs (Fig. 3a), so the AQP5^+^ cells were not the typical acinar cells in morphology. The LG acini culture required fetal bovine serum (FBS) [29], and the addition of FBS could enhance the secretion of acini in vitro [30]. Also, FBS could enable the differentiation of stem cells [31]. Therefore, FBS was added to induce acinar differentiation of LGSCs. After 14-day culture in the FBS condition, there were low nucleo-cytoplasmic ratio cells inside the LGSC spheres, similar to acinar cells (Fig. 3c, d), highly expressing AQP5 (Fig. 3e). In addition, these acinar-like cells did not show any expression of apoptotic markers (Additional file 5: Figure S2 E). These data indicated that LGSCs could differentiate into acinar-like cells by the induction of serum. Therefore, the 3D culture condition for LGSC differentiation was confirmed.

### LGSCs improved symptoms of ADDED

To test whether LGSCs could restore function in diseased LGs, SjS mouse model—NOD/ShiLtJ mice were used as recipients in which there is a chronic autoimmune attack against LGs resulting in ADDED [32]. Wide range lymphocytic infiltration could be detected in the NOD/ShiLtJ mouse LGs after 12 weeks (Additional file 6: Figure S3 A–C), and the tear secretion of NOD/ShiLtJ mice was significantly decreased (Additional file 6: Figure S3 D). The inflammation and decay in NOD/ShiLtJ mice orbits became more severe as they became older (Additional file 6: Figure S3 E). Hence, all the recipient mice used for LGSC transplantation were older than 12 weeks.

For tracking the cells injected into mice, LGSCs from transgenic mice—ROSA26^mT/mG^ mice were serum-free cultured as donor cells for transplantation and designated ROSA-LGSCs (Additional file 7: Figure S4 A). ROSA-LGSCs also expressed the stem/progenitor cell markers Krt5, Krt14, P63, and nestin (Additional file 7: Figure S4 B) and could differentiate into acinar and ductal cells as well (Additional file 7: Figure S4 C, D), suggesting that ROSA-LGSCs had the same characteristics as LGSCs.

**Fig. 4:**
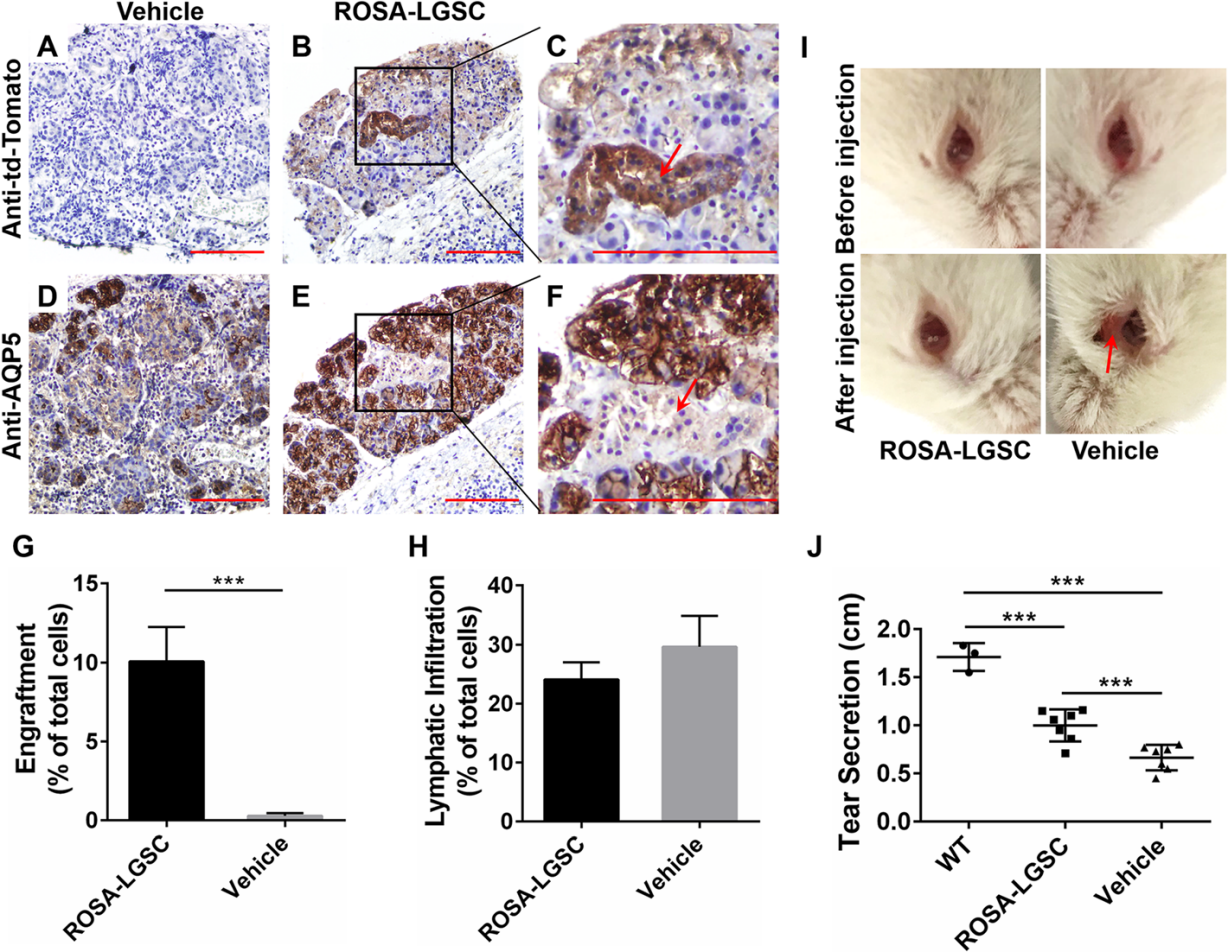
Engraftment of LGSC allotransplantation and relief of ADDED symptoms. **a–f** Immunohistochemistry (IHC) staining of NOD/ShiLtJ LG transplanted with ROSA-LGSCs cultured for 7 days after 8 weeks. **a–c** IHC staining with anti-td-Tomato antibody; **d–f** IHC staining with anti-AQP5 antibody; **a, d** LG injected with vehicle, **b, e** LG injected with ROSA-LGSCs, **c, f** the magnified image of the black frame in **b, e** (red arrow, intralobular duct); scale bar, 100 μm. **g** The ratio between td-Tomato-positive cell areas and total cell areas of transplanted LGs. *n* = 7; ***, *P* < 0.01. **h** The ratio between lymphatic infiltration areas and total cell areas of transplanted LGs. *n* = 7; ***, *P* = 0.3718. **i** The condition of the NOD/ShiLtJ mice eye orbit before and after injection of ROSA-LGSCs at 8 weeks. There is no decay of the eye orbit on the cell-injected side, whereas there is obvious decay of the eye orbit (red arrow) on the control side. **j** Tear volume of wild type and NOD/ShiLtJ mice transplanted with ROSA-LGSCs cultured for 7 days after 8 weeks. The tear volume of ROSA-LGSC-injected LGs is significantly higher compared to the control LGs, whereas it is significantly lower compared to WT LGs. WT, wild type, *n* = 3; NOD/ShiLtJ mice, *n* = 7; ***, P < 0.01

LGSC transplantation was performed by orthotopic injection of ROSA-LGSCs cultured for 7 or 14 days, representing the population of stem cells without differentiation or the cells in different differentiation stages (Fig. 2e, f), respectively. All the ROSA-LGSCs used as donor cells were passaged at least five times. After being digested into single cells, ROSA-LGSCs were injected into recipient LGs on one side, while the vehicle was injected into the contralateral LGs as a control. We harvested the subjects for detection in 8 weeks, and there was no carcinogenesis in all recipient mice. We studied the subjects that were injected with ROSA-LGSCs cultured for 7 days. Immunohistochemistry (IHC) analysis showed that new lacrimal lobules were derived from ROSA-LGSCs in some cell-injected LGs (Fig. 4a–c) and they were composed of mature acinar cells (AQP5^high^) as well as intralobular duct (AQP5^low^) (Fig. 4d–f). There were no obvious infiltration foci in the new lobules. Interestingly, we also found that the ROSA-LGSCs could engraft around the infiltration foci and differentiate into the acini and ducts (Additional file 8: Figure S5 D–F). The td-Tomato-positive areas and lymphatic infiltration areas were quantified to estimate the engraftment efficiency and anti-inflammation effect of ROSA-LGSCs, respectively. The results showed that the engraftment efficiency of ROSA-LGSCs was approximate 10% and the area of lymphatic infiltration was decreased (Fig. 4g, h). The inflammation and decay of the orbit on the cell-injected side were significantly improved compared to the control side (Fig. 4i). For assessing functional recovery, we measured the tear volume of the recipient mice on each side using phenol red-stained cotton threads. Statistical analysis indicated that the tear secretion of ROSA-LGSC-injected LGs was significantly increased compared to the control LGs, whereas it did not fully recover to the normal level compared to wild type mice (Fig. 4j). Overall, these data indicated that LGSCs could engraft into diseased LGs and differentiate into acinar and ductal cells to recover the secretory function of LGs partially.

We then performed the same detection procedure on the mice that were injected with ROSA-LGSCs cultured for 14 days. Nevertheless, there was no engraftment in the LGs (Table 1), suggesting the intermediate differentiated cells could not restore the function of diseased LGs.

**Table 1:** The success ratio of treatment by ROSA-LGSCs for different culture times

Culture time of ROSA-LGSCs (days)	Cell number for injection	Mice number of allograft	Mice number of engraftment	Success ratio of engraftment
7	2 × 10^4^	16	7	43.75%
14	2 × 10^4^	12	0	0

ROSA-LGSCs cultured for 7 or 14 days were used for the treatment of aqueous-deficient dry eye disease (ADDED) mice. The success ratio of engraftment of ROSA-LGSCs cultured for 7 days was much higher than ROSA-LGSCs cultured for 14 days

### LGSCs isolated from diseased mice repaired the injured LGs

As allogeneic stem cell transplantation could cause graft-versus-host disease (GvHD), which results in immune attacks against the tissue of the recipients [33], we attempted to isolate the LGSCs from diseased LGs using the same 3D culture method that we established to evaluate the feasibility of autotransplantation. LGSCs were obtained from NOD/ShiLtJ mice that were over 12 weeks old and were named NOD-LGSCs (Additional file 9: Figure S6 A), whereas the number of NOD-LGSC spheres was significantly less than the LGSC spheres in primary culture (Additional file 9: Figure S6 B, C). However, despite this, NOD-LGSCs also expressed stem cell markers Krt14, Krt5, P63, and nestin (Additional file 9: Figure S6 D). AQP5 and Ltf were significantly upregulated in NOD-LGSCs cultured for 14 days compared to LGSC spheres cultured for 5 and 7 days, whereas the expression of stem cell maker Krt14 was significantly downregulated (Additional file 9: Figure S6 E). In addition, IF analysis also demonstrated that the cells expressing Krt14 and Ki67 were significantly decreased and mostly located in the outer layers of LGSC spheres (Additional file 9: Figure S6 F). However, unlike LGSCs, the ductal marker gene Krt19 was not upregulated and Krt19^+^ cells did not arise from the inner layers of NOD-LGSC spheres (Additional file 9: Figure S6 E, F). These results suggested that NOD-LGSCs possessed similar characteristics to LGSCs, but were not as capable as LGSCs in self-renewal and differentiation.

**Fig. 5:**
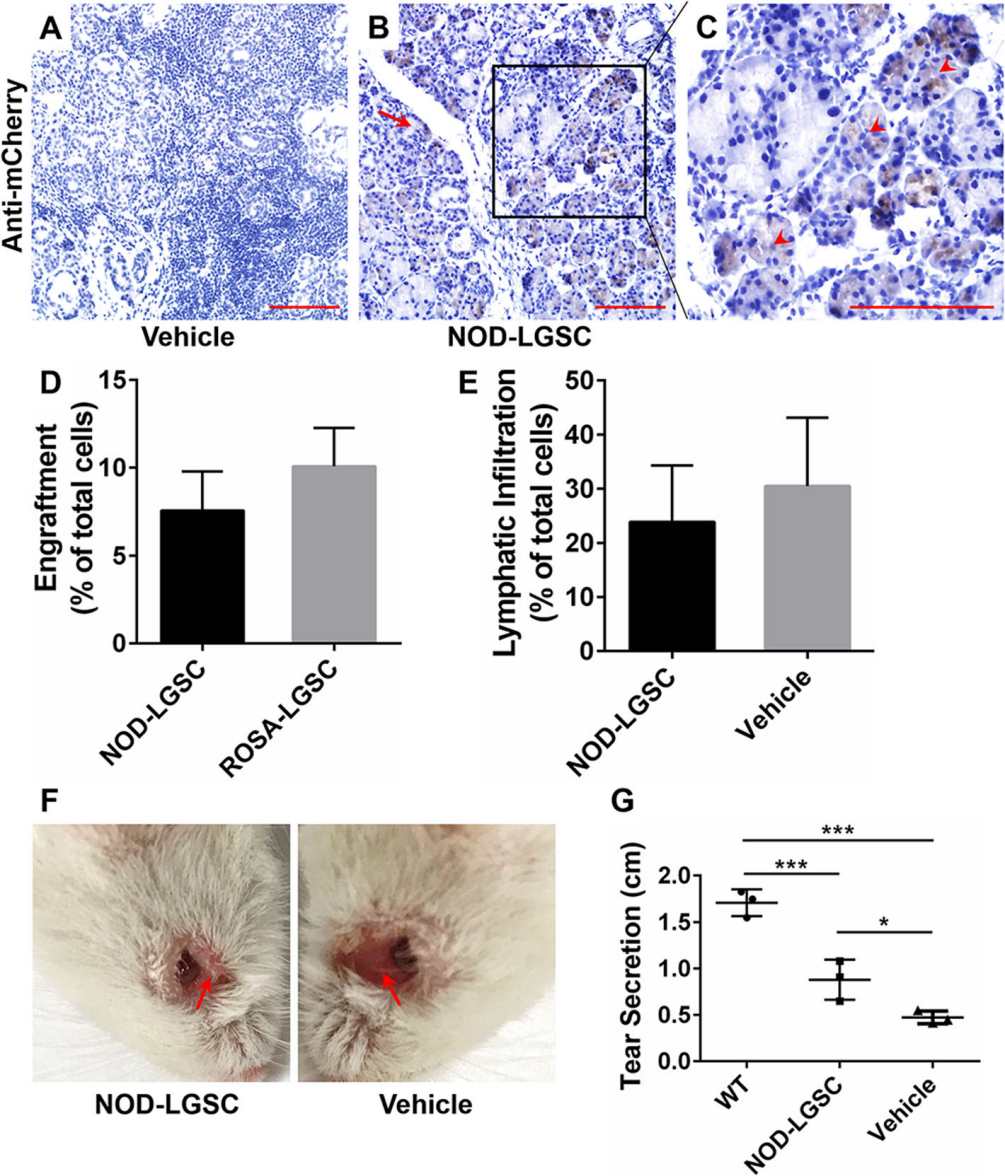
Engraftment of NOD-LGSC transplantation and slight relief of ADDED symptoms. **a–c** IHC staining with anti-mCherry antibody of NOD/ShiLtJ LG transplanted with NOD-LGSCs cultured for 7 days after 8 weeks. **a** LG injected with vehicle, **b** LG injected with NOD-LGSCs (red arrow, intralobular duct), **c** the magnified image of the black frame in **b** (red arrowhead, acini); scale bar, 100 μm. **d** The ratio between td-Tomato-positive cell areas and total cell areas of NOD-LGSCs and ROSA-LGSCs transplanted LGs. ROSA-LGSCs transplanted LGs, *n* = 7; NOD-LGSCs transplanted LGs, *n* = 3; *P* = 0.5180. **e** The ratio between lymphatic infiltration areas and total cell areas of NOD-LGSCs transplanted LGs. *n* = 3, *P* = 0.7084. **f** The condition of the NOD/ShiLtJ mice eye orbit at 8 weeks after injection of NOD-LGSCs. The decay of the eye orbit (red arrow) on the cell-injected side is improved compared to the control side. **g** Tear volume of wild type and NOD/ShiLtJ mice transplanted with NOD-LGSCs cultured for 7 days after 8 weeks. The tear volume of ROSA-LGSC-injected LGs is significantly higher compared to the control LGs, whereas it is significantly lower compared to WT LGs. WT, wild type, *n* = 3; NOD/ShiLtJ mice, *n* = 3; ***, *P* < 0.01; *, *P* < 0.05

NOD-LGSCs were then labeled with mCherry fluorescent protein (Additional file 9: Figure S6 G) and transplanted by the strategy that we developed (injecting 2 × 10^4^ cells cultured for 7 days and harvesting subjects after 8 weeks). IHC analysis showed that NOD-LGSCs engrafted in the diseased LGs and generated the acini and intralobular ducts (Fig. 5a–c). However, the success ratio of engraftment and engraftment efficiency of NOD-LGSC were lower than these of ROSA-LGSCs (Table 2, Fig. 5d). The lymphatic infiltration was also decreased similar to the engraftment of ROSA-LGSCs (Fig. 5e). The inflammation and decay of the orbit were eased, and the tear secretion was also significantly improved after NOD-LGSCs injection (Fig. 5f, g). These results revealed that the LGSCs residing in the diseased LGs (NOD-LGSCs) could be cultured and expanded using our culture strategy, NOD-LGSCs could still engraft into the diseased LGs and relieved the ADDED symptoms.

**Table 2:** The success ratio of treatment by ROSA-LGSCs and NOD-LGSCs

Type of LGSCs	Mice number of orthotopic injection	Mice number of engraftment	Success ratio of engraftment
ROSA-LGSCs	16	7	43.75%
NOD-LGSCs	12	3	25%

The success ratio of treatment by NOD-LGSCs is lower than ROSA-LGSCs

## Discussion

LG regeneration and self-healing depend on the stem/progenitor cells residing in the niche. It is a vital prerequisite for research and clinical application for DED to establish a long-term culture of LG stem/progenitor cells [34]. Previous study has shown successful isolation and culture of adult mouse LG epithelial cells by 2D culture with fetal calf serum, which express stem cell markers such as oct4, sox2, nanog, and nestin [35]. However, these cells cannot be defined as LG stem cells as they do not possess the ability to generate the acini and ducts in vivo to repair the LG injury. Further, the presence of serum in LGSC culture is not suitable for transplantation. Our study demonstrated a serum-free 3D culture protocol for LG stem cell isolation and long-term expansion from mice LGs. The isolated stem cell population, LGSCs, could express the stem/progenitor cell markers Krt14, Krt5, P63, and nestin and restore the secretory function of diseased LGs.

Several signaling pathways are crucial for the development of the LG. The EGFR/ErbB signaling pathway activated by EGF could promote epithelial stem cell proliferation [12, 36]. The FGFR2b signaling pathway activated by FGF10 regulates LG stem/progenitor cell proliferation and the branching morphogenesis of the LG bud [27, 37]. In addition, canonical Wnt signaling is involved in the regulation of FGF10 in LG development and it is essential for maintenance of stem cells [38, 39]. We analyzed the RNA-sequence of embryonic LGs and found that the EGFR/ErbB signaling pathway and canonical Wnt signaling pathway were significantly activated, accompanied by FGF10 expressed at a very high level (data not shown). What is more, Y-27632 has been shown to delay the senescence of stem cells in vitro [40]. Hence, EGF, FGF10, Wnt3A, and Y-27632 were added into the medium for the LGSC culture. In this study, we found that EGF, FGF10, and Y-27632 were indispensable to the LGSC culture. Wnt3A was necessary for the LGSC passage culture, instead of the primary culture, suggesting the regulatory effect of Wnt signaling on self-renewal and proliferation of the LGSCs in a continuous passage culture in vitro. The results from salivary gland stem cell (SGSC) culture also confirmed our finding [24].

The potential of differentiation in vitro is one of the characteristics that define stem cells. Pluripotent stem cells can differentiate into cells from all three germ layers [41], and the adult tissue-specific stem cells can also differentiate and form organoids in vitro, such as the pancreas [21], mammary gland [22], salivary gland [23, 24], and intestine [42]. In LG development, the interaction between the epithelial bud and surrounding mesenchyme plays a dominant role and depends on the degradation of the ECM [27], by which the decrease of the matrix rigidity surrounding the epithelial bud is accompanied. In our study, reducing the rigidity of Matrigel (1/3 concentration) could induce LGSCs differentiating into the Krt19^+^ ductal-like cells located in the inner layers of the LGSC sphere, while the residual Krt14^+^ cells located in the outer layers were similar to the structure of suprabasal cells and basal cells in the LG duct, suggesting that the biophysical microenvironment was important for ductal differentiation of LGSCs. Furthermore, FBS was essential for the survival of the LG and mammary gland acini in vitro [29, 43] and was used for the differentiation of epithelial cells and stem cells [31, 44]. In this study, we showed that adding FBS to the medium could induce the LGSCs differentiating into acinar-like cells that highly expressed AQP5, suggesting that unknown factors in the complex FBS promoted acinar differentiation of LGSCs. These results indicated that the LGSCs possessed the feature of differentiation in vitro*,* supporting LGSCs as the stem cells of the adult LG. Further research is needed to determine the mechanism underlying LGSC differentiation in vitro.

Previous studies have reported that mesenchymal stem cell (MSC) therapy is an alternative strategy for treatment of DED, which could suppress inflammation and increase tear production [45, 46]. As several reports have described MSCs transplanted by vein injection [47, 48], we first attempted to transplant the LGSCs into NOD/ShiLtJ mice by tail vein injection. After 10 days, the LGSCs engrafted the diseased LGs rather than other organs such as the salivary gland, pancreas, or liver (Additional file 8: Figure S5 A–C), suggesting that the engraftment of LGSCs were specific to LGs. However, the tail vein injected LGSCs did not survive to generate acinar and ductal cells. For this reason, we then carried out the intraglandular injection for LGSC transplantation. Similar to EPCP transplantation into *TSP-1*^*−/−*^ mouse LGs [9], the LGSCs could engraft into the NOD/ShiLtJ mouse LG and partially restore secretory function. Our results showed two rescue approaches for the LGSCs: (1) directly integrating into the injured lobules and restoring the primary structure and (2) engrafting at the interlobular site and generating nascent lobules, composed of acini and intralobular ducts. In both conditions, the infiltration foci mainly decreased in the recovery areas, corresponding to the study of EPCPs [9]. We concluded two main hypotheses to explain the anti-inflammation effect. The first, LGSCs might secrete growth factors, which could suppress the lymphatic infiltration. The second, LGSCs from the donor differentiated into acini and ducts and repaired the damage of diseased LG temporarily, as we just detected the post-transplantation LGs after 8 weeks. Hence, further study is needed to be performed like analyses of LGSC secretory protein and tracing the repairment of diseased LGs after transplantation for longer time. As the LGSCs showed a dynamic change related to the culture time, we compared the success rate of the LGSC engraftment in differentiation conditions and confirmed the allotransplantation of 2 × 10^4^ LGSCs cultured for 7 days was the optimal strategy for DED treatment with our strategy.

Additionally, we demonstrated that the culture protocol that we established could also be applied to the isolation and long-term expansion of the LGSCs from diseased LGs (NOD-LGSCs). NOD-LGSCs could repair the diseased LGs after transplantation by the confirmed optimal strategy, indicating the feasibility of LGSC autotransplantation. The lymphatic infiltration was suppressed similar to the repairment of ROSA-LGSCs, and the acini or intralobular ducts derived from diseased LG emerged in the recovery area as well, suggesting that NOD-LGSCs might promote the differentiation of stem cells residing in the diseased LG simultaneously. However, the efficiency of NOD-LGSC engraftment was lower than LGSC engraftment, and the ductal marker Krt19 was not upregulated when prolonging the culture time in vitro. These results indicated that the pathological microenvironment might decrease the stem cell potentials of NOD-LGSCs. The mechanism for the difference between LGSCs and NOD-LGSCs needs to be further explored, which may help to recover the stem cell capacity of ADDED LGs and improve engrafting efficiency.

## Conclusion

In summary, our study demonstrated a robust serum-free 3D culture strategy for the isolation of adult LGSCs. LGSCs possess the capacity of self-renewal, long-term expansion, and differentiation into acinar and ductal cells in vitro and in vivo. The LGSCs could improve the secretory function of diseased LGs. Furthermore, NOD-LGSCs from diseased LGs could also engraft into the diseased LGs. Hence, our work provides a perspective for individualized stem cell therapy of ADDED in future clinical applications.

## Supplementary information

Additional file 1: **Table S1.** The list of primary antibodies. All the primary antibodies used in this study. Table S2. The list of fluorescent secondary antibodies. All the flourescent secondary antibodies used in this study (XLS 33 kb)

Additional file 2: **Table S3.** Sequences of primers. All the sequences of primers used in this study (XLS 27 kb)

Additional file 3: **Table S4.** Differential expressed gene list of LGSCs cultured 7 days and 14 days. All the genes expressed differentially between day 7 LGSCs and day 14 LGSCs (XLS 44 kb)

Additional file 4: **Figure S1.** Optimization of lacrimal gland stem cell medium (LGSCM). A. The morphology of primary cultured LGSCs at day 7 in the LGSCM and withdrawing of EGF, FGF10, Wnt3A, and Y-27632, respectively. B. The diameter of primary cultured LGSCs at day 7 in the LGSCM and withdrawing of EGF, FGF10, Wnt3A, and Y-27632, respectively. C. The cell numbers of primary cultured LGSCs at day 7 in the LGSCM and withdrawing of EGF, FGF10, Wnt3A, and Y-27632, respectively. D. The morphology of passaged LGSCs at day 7 in the LGSCM and withdrawing of Wnt3A. E. The diameter of passaged LGSCs at day 7 in the LGSCM and withdrawing of Wnt3A. F. The cell numbers of passaged LGSCs at day 7 in the LGSCM and withdrawing of Wnt3A. (PDF 7184 kb)

Additional file 5: **Figure S2.** Characterization of LGSCs cultured in different time. A. Immuno-fluorescent staining of LGCSs cultured for 7 days. Epcam (red, epithelial cell marker), VEGFR2 (green, endothelial cell marker), FAP-α (green, fibroblast marker), scale bar, 50 μm. Nuclear staining, DAPI (blue). B. The morphology of day 7 LGSCs subcultured from LGSCs cultured for 7 days; scale bar, 400 μm. C. The morphology of day 7 LGSCs subcultured from LGSCs cultured for 14 days; scale bar, 400 μm. D. The sphere number per-field of LGSCs. L7, LGSCs derived from LGSCs cultured for 7 days; L14, LGSCs derived from LGSCs cultured for 14 days; ***, *P* < 0.01; *n* = 5. E. Immuno-fluorescent staining of LGCSs cultured for 14 days. E-cadherin (red, epithelial cell marker), Caspase-3 (green, apoptosis marker), Caspase-7 (green, apoptosis marker), scale bar, 50 μm. Nuclear staining, DAPI (blue) (PDF 16604 kb)

Additional file 6: **Figure S3.** Examination of dry eye symptoms of NOD/ShiLtJ mice. A–C. Hematoxylin and eosin staining of 12-week wild type (A) and NOD/ShiLtJ (B, C) mice LG; WT, wild type; scale bar,100 μm. D. Tear volume of 12-week wild type and NOD/ShiLtJ mice; WT, wild type; ***, *P* < 0.01; *n* = 3. E. The condition of 12-week and 36-week NOD/ShiLtJ mice eye orbits (red arrow, decay of the eye orbit) (PDF 2809 kb)

Additional file 7: **Figure S4.** Isolation and characterization of ROSA-LGSGs. A. The fluorescent images of primary cultured NOD-LGSCs at day 7; BF, bright field. B. Gene expression of adult stem/progenitor cell markers of LGSCs and ROSA-LGSCs. C. Gene expression of adult stem cell and differentiated markers of ROSA-LGSCs cultured for 5, 7, 10, and 14 days. AQP5, marker of secretory cells; Ltf, secretory protein gene of LG; Krt19, marker of ductal cells; Krt14, marker of adult stem cells; ***, *P* < 0.01; *n* = 3. D. Immunofluorescent staining of ROSA-LGCSs cultured for 7 and 14 days. Krt14 (red, stem cells), Krt19 (red, ductal cells), Ki67 (red, proliferative cells), scale bar, 50 μm. Nuclear staining, DAPI (blue) (PDF 9434 kb)

Additional file 8: **Figure S5.** Engraftment of LGSC allotransplantation. A. The fluorescent images of NOD/ShiLtJ organs at 10 days post tail vein injection of ROSA-LGSCs. LG, lacrimal gland; SG, salivary gland. B, C. Immunofluorescent staining with anti-td-Tomato antibody of NOD/ShiLtJ LG tail vein injected with ROSA-LGSCs cultured for 7 days after 10 days. B. LG injected with ROSA-LGSCs (white arrow, green, exogenous cells). C. LG injected with vehicle; scale bar, 100 μm. D–F. IHC staining with anti-td-Tomato antibody of NOD/ShiLtJ LG transplanted with ROSA-LGSCs cultured for 7 days after 8 weeks. D. LG injected with vehicle. E. LG injected with ROSA-LGSCs. F. the magnified image of the black frame in E (red arrow, intra-lobular duct; red arrowhead, acini); scale bar, 100 μm (PDF 10480 kb)

Additional file 9: **Figure S6.** Isolation and characterization of NOD-LGSGs. A, B. The morphology of LGSCs and NOD-LGSCs in primary culture at day 7; scale bar, 400 μm. A. NOD-LGSCs; B. LGSCs. C. The sphere number per-field of NOD-LGSCs is significantly less than LGSCs. Scale bar, 400 μm; ***, P < 0.01; *n* = 5. D. Transcriptional expression of adult stem/progenitor cell markers of LGSCs and NOD-LGSCs. NC, negative control. E. Transcriptional expression of adult stem cell and differentiated markers of NOD-LGSCs cultured for 5, 7, 10, and 14 days. Secretory cell marker AQP5 and secretory protein gene of LG Ltf are significantly up-regulated. Adult stem cell marker Krt14 is significantly down-regulated. There is no significant change of ductal cell marker Krt19; ***, P < 0.01; ns, non-significance; n = 3. F. Immunofluorescent staining of NOD-LGCSs cultured for 5, 7, 10, and 14 days. As elongating culture time, cells expressing Krt14 (red) and Ki67 (red) are significantly decreased, and no cells expressing Krt19 (red) emerge, scale bar, 50 μm. Nuclear staining, DAPI (blue). G. The fluorescent images of NOD-LGSCs cultured for 7 days after being labeled with mCherry. BF, bright field (PDF 9808 kb)

## Abbreviations

ADDED: Aqueous-deficient dry eye disease
AQP: Aquaporin
DAPI: 4′,6-Diamidino-2-phenylindole
DED: Dry eye disease
DMEM: Dulbecco’s modified Eagle medium
EGF: Epidermal growth factor
FACS: Fluorescence-activated cell sorting
FBS: Fetal bovine serum
FGF: Fibroblast growth factor
H&E: Hematoxylin and eosin
IF: Immunofluorescence
IHC: Immunohistochemistry
Krt: Keratin
LG: Lacrimal gland
LGSC: Lacrimal gland stem cell
LGSCM: Lacrimal gland stem cell medium
Ltf: Lactotransferrin
qRT-PCR: Quantitative reverse transcription polymerase chain reaction
RT-PCR: Reverse transcription polymerase chain reaction
