# Supplementary material for: Establishment of long-term serum-free culture for lacrimal gland stem cells aiming at lacrimal gland repair
Source: Stem Cell Res Ther. 2020 Jan 8;11:20. doi: 10.1186/s13287-019-1541-1 (PMC6951017; doi:10.1186/s13287-019-1541-1)
Supplement: Supplementary file 4 — Figure S1. Optimization of lacrimal gland stem cell medium (LGSCM). A. The morphology of primary cultured LGSCs at day 7 in the LGSCM and withdrawing of EGF, FGF10, Wnt3A, and Y-27632, respectively. B. The diameter of primary cultured LGSCs at day 7 in the LGSCM and withdrawing of EGF, FGF10, Wnt3A, and Y-27632, respectively. C. The cell numbers of primary cultured LGSCs at day 7 in the LGSCM and withdrawing of EGF, FGF10, Wnt3A, and Y-27632, respectively. D. The morphology of passaged LGSCs at day 7 in the LGSCM and withdrawing of Wnt3A. E. The diameter of passaged LGSCs at day 7 in the LGSCM and withdrawing of Wnt3A. F. The cell numbers of passaged LGSCs at day 7 in the LGSCM and withdrawing of Wnt3A. (PDF 7184 kb) [file 13287_2019_1541_MOESM4_ESM.pdf]

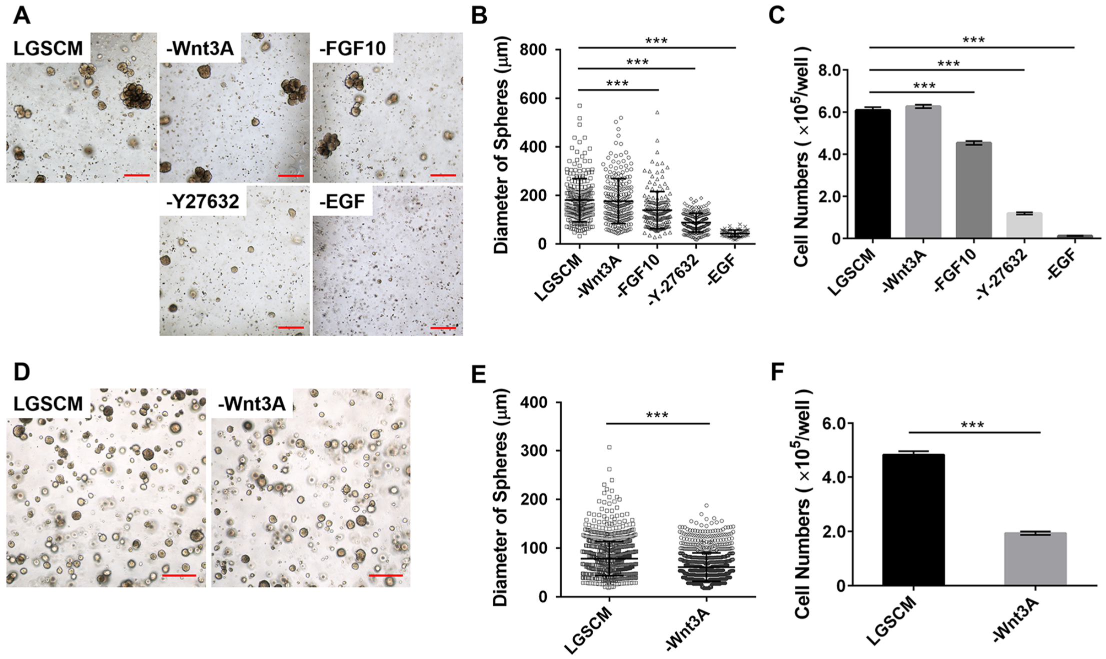

Figure S1. Optimization of lacrimal gland stem cell medium (LGSCM). A. The morphology of primary cultured LGSCs at day 7 in the LGSCM and withdrawing of EGF, FGF10, Wnt3A, and Y-27632, respectively. B. The diameter of primary cultured LGSCs at day 7 in the LGSCM and withdrawing of EGF, FGF10, Wnt3A, and Y-27632, respectively. C. The cell numbers of primary cultured LGSCs at day 7 in the LGSCM and withdrawing of EGF, FGF10, Wnt3A, and Y-27632, respectively. D. The morphology of passaged LGSCs at day 7 in the LGSCM and withdrawing of Wnt3A. E. The diameter of passaged LGSCs at day 7 in the LGSCM and withdrawing of Wnt3A. F. The cell numbers of passaged LGSCs at day 7 in the LGSCM and withdrawing of Wnt3A.
