# Supplementary material for: Establishment of long-term serum-free culture for lacrimal gland stem cells aiming at lacrimal gland repair
Source: Stem Cell Res Ther. 2020 Jan 8;11:20. doi: 10.1186/s13287-019-1541-1 (PMC6951017; doi:10.1186/s13287-019-1541-1)
Supplement: Supplementary file 5 — Figure S2. Characterization of LGSCs cultured in different time. A. Immuno-fluorescent staining of LGCSs cultured for 7 days. Epcam (red, epithelial cell marker), VEGFR2 (green, endothelial cell marker), FAP-α (green, fibroblast marker), scale bar, 50 μm. Nuclear staining, DAPI (blue). B. The morphology of day 7 LGSCs subcultured from LGSCs cultured for 7 days; scale bar, 400 μm. C. The morphology of day 7 LGSCs subcultured from LGSCs cultured for 14 days; scale bar, 400 μm. D. The sphere number per-field of LGSCs. L7, LGSCs derived from LGSCs cultured for 7 days; L14, LGSCs derived from LGSCs cultured for 14 days; ***, P < 0.01; n = 5. E. Immuno-fluorescent staining of LGCSs cultured for 14 days. E-cadherin (red, epithelial cell marker), Caspase-3 (green, apoptosis marker), Caspase-7 (green, apoptosis marker), scale bar, 50 μm. Nuclear staining, DAPI (blue) (PDF 16604 kb) [file 13287_2019_1541_MOESM5_ESM.pdf]

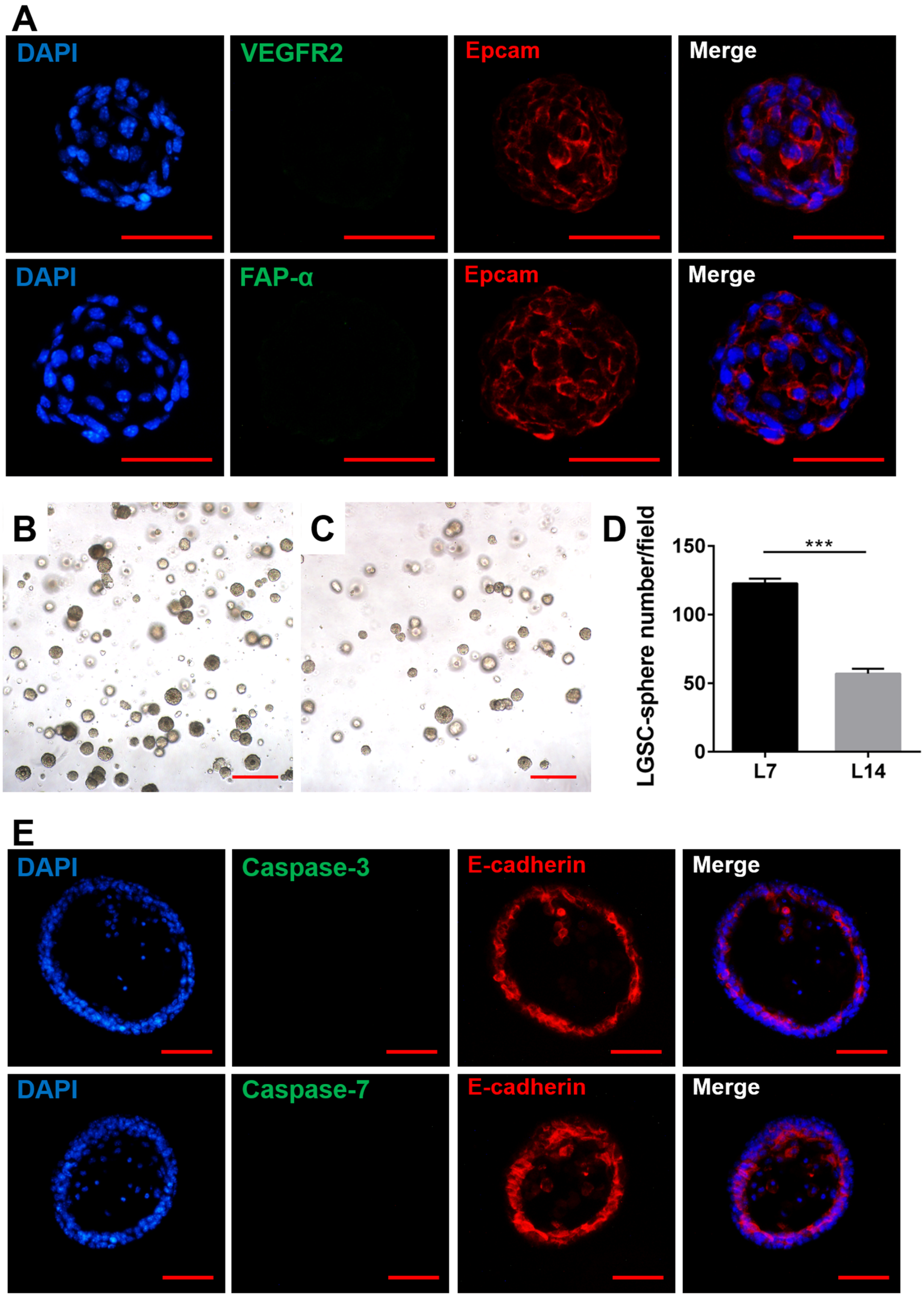

Figure S2. Characterization of LGSCs cultured in different time. A. Immuno-fluorescent staining of LGCSs cultured for 7 days. Epcam (red, epithelial cell marker), VEGFR2 (green, endothelial cell marker), FAP-α (green, fibroblast marker), scale bar, 50 μm. Nuclear staining, DAPI (blue). B. The morphology of day 7 LGSCs subcultured from LGSCs cultured for 7 days; scale bar, 400 μm. C. The morphology of day 7 LGSCs subcultured from LGSCs cultured for 14 days; scale bar, 400 μm. D. The sphere number per-field of LGSCs. L7, LGSCs derived

from LGSCs cultured for 7 days; L14, LGSCs derived from LGSCs cultured for 14 days; ***, $P < 0.01$; $n = 5$. E. Immuno-fluorescent staining of LGCSs cultured for 14 days. E-cadherin (red, epithelial cell marker), Caspase-3 (green, apoptosis marker), Caspase-7 (green, apoptosis marker), scale bar, 50 μm. Nuclear staining, DAPI (blue).
