# Supplementary material for: Establishment of long-term serum-free culture for lacrimal gland stem cells aiming at lacrimal gland repair
Source: Stem Cell Res Ther. 2020 Jan 8;11:20. doi: 10.1186/s13287-019-1541-1 (PMC6951017; doi:10.1186/s13287-019-1541-1)
Supplement: Supplementary file 6 — Figure S3. Examination of dry eye symptoms of NOD/ShiLtJ mice. A–C. Hematoxylin and eosin staining of 12-week wild type (A) and NOD/ShiLtJ (B, C) mice LG; WT, wild type; scale bar,100 μm. D. Tear volume of 12-week wild type and NOD/ShiLtJ mice; WT, wild type; ***, P < 0.01; n = 3. E. The condition of 12-week and 36-week NOD/ShiLtJ mice eye orbits (red arrow, decay of the eye orbit) (PDF 2809 kb) [file 13287_2019_1541_MOESM6_ESM.pdf]

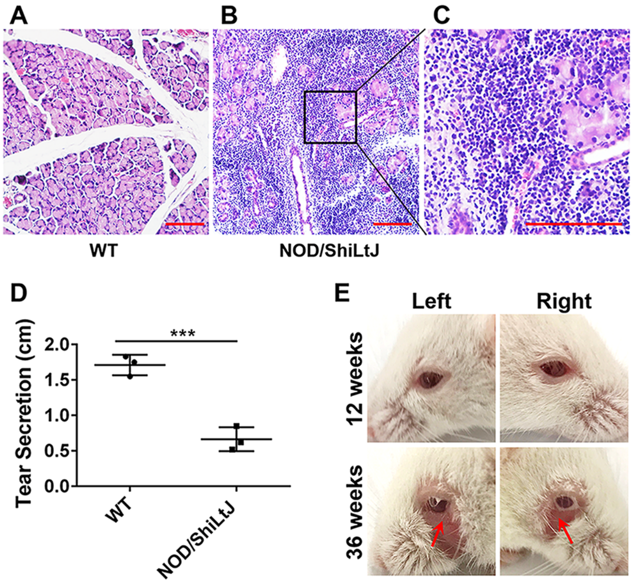

Figure S2. Examination of dry eye symptoms of NOD/ShiLtJ mice**.** A–C. Hematoxylin and eosin staining of 12-week wild type (A) and NOD/ShiLtJ (B, C) mice LG; WT, wild type; scale bar,100 µm. D. Tear volume of 12-week wild type and NOD/ShiLtJ mice; WT, wild type; ***, $P < 0.01$; $n = 3$. E. The condition of 12-week and 36-week NOD/ShiLtJ mice eye orbits (red arrow, decay of the eye orbit).
