# Supplementary material for: Establishment of long-term serum-free culture for lacrimal gland stem cells aiming at lacrimal gland repair
Source: Stem Cell Res Ther. 2020 Jan 8;11:20. doi: 10.1186/s13287-019-1541-1 (PMC6951017; doi:10.1186/s13287-019-1541-1)
Supplement: Supplementary file 7 — Figure S4. Isolation and characterization of ROSA-LGSGs. A. The fluorescent images of primary cultured NOD-LGSCs at day 7; BF, bright field. B. Gene expression of adult stem/progenitor cell markers of LGSCs and ROSA-LGSCs. C. Gene expression of adult stem cell and differentiated markers of ROSA-LGSCs cultured for 5, 7, 10, and 14 days. AQP5, marker of secretory cells; Ltf, secretory protein gene of LG; Krt19, marker of ductal cells; Krt14, marker of adult stem cells; ***, P < 0.01; n = 3. D. Immunofluorescent staining of ROSA-LGCSs cultured for 7 and 14 days. Krt14 (red, stem cells), Krt19 (red, ductal cells), Ki67 (red, proliferative cells), scale bar, 50 μm. Nuclear staining, DAPI (blue) (PDF 9434 kb) [file 13287_2019_1541_MOESM7_ESM.pdf]

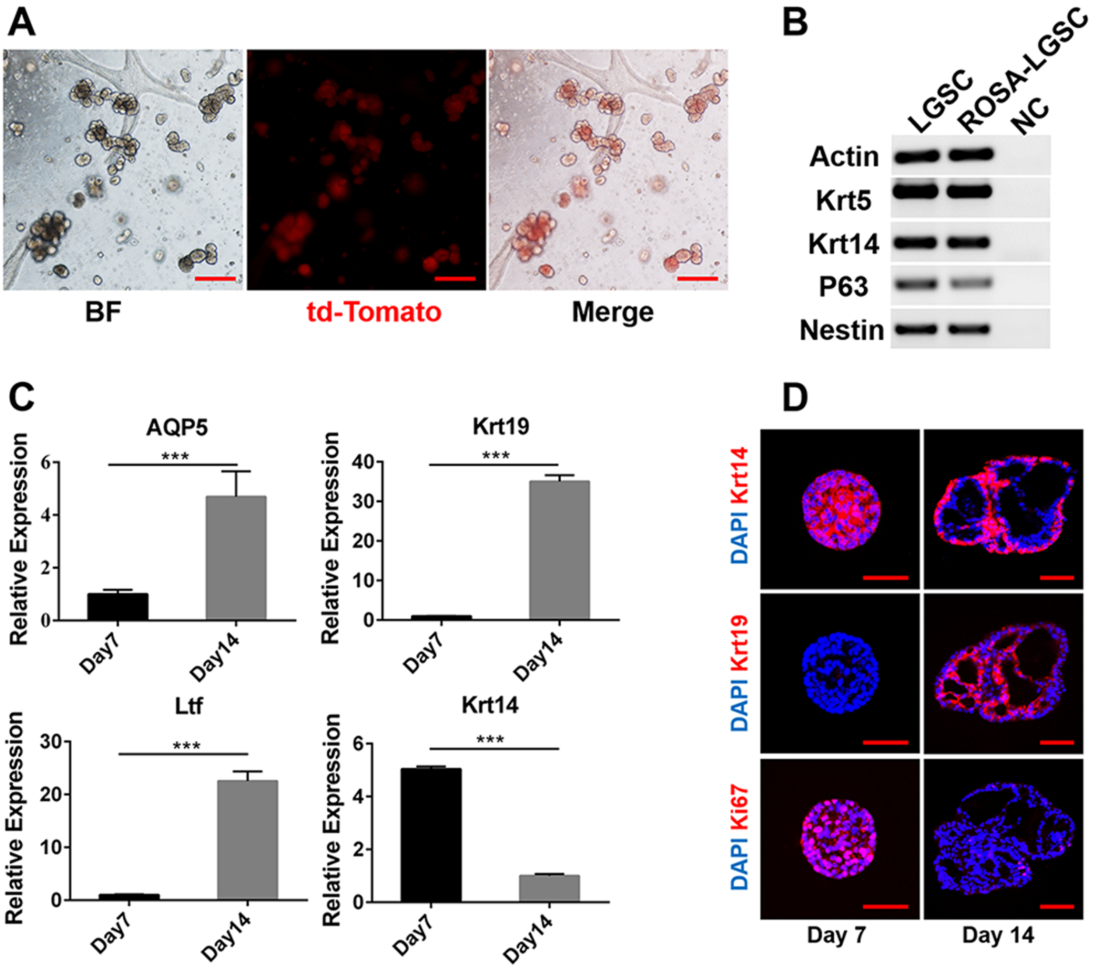

Figure S3. Isolation and characterization of ROSA-LGSGs. A. The fluorescent images of primary cultured NOD-LGSCs at day 7; BF, bright field. B. Gene expression of adult stem/progenitor cell markers of LGSCs and ROSA-LGSCs. C. Gene expression of adult stem cell and differentiated markers of ROSA-LGSCs cultured for 5, 7, 10, and 14 days. AQP5, marker of secretory cells; Ltf, secretory protein gene of LG; Krt19, marker of ductal cells; Krt14, marker of adult stem cells; ***, P < 0.01; n = 3. D. Immunofluorescent staining of ROSA-LGCSs cultured for 7 and 14 days. Krt14 (red, stem cells), Krt19 (red, ductal cells), Ki67 (red, proliferative cells), scale bar, 50 μm. Nuclear staining, DAPI (blue).
