# Supplementary material for: Establishment of long-term serum-free culture for lacrimal gland stem cells aiming at lacrimal gland repair
Source: Stem Cell Res Ther. 2020 Jan 8;11:20. doi: 10.1186/s13287-019-1541-1 (PMC6951017; doi:10.1186/s13287-019-1541-1)
Supplement: Supplementary file 8 — Figure S5. Engraftment of LGSC allotransplantation. A. The fluorescent images of NOD/ShiLtJ organs at 10 days post tail vein injection of ROSA-LGSCs. LG, lacrimal gland; SG, salivary gland. B, C. Immunofluorescent staining with anti-td-Tomato antibody of NOD/ShiLtJ LG tail vein injected with ROSA-LGSCs cultured for 7 days after 10 days. B. LG injected with ROSA-LGSCs (white arrow, green, exogenous cells). C. LG injected with vehicle; scale bar, 100 μm. D–F. IHC staining with anti-td-Tomato antibody of NOD/ShiLtJ LG transplanted with ROSA-LGSCs cultured for 7 days after 8 weeks. D. LG injected with vehicle. E. LG injected with ROSA-LGSCs. F. the magnified image of the black frame in E (red arrow, intra-lobular duct; red arrowhead, acini); scale bar, 100 μm (PDF 10480 kb) [file 13287_2019_1541_MOESM8_ESM.pdf]

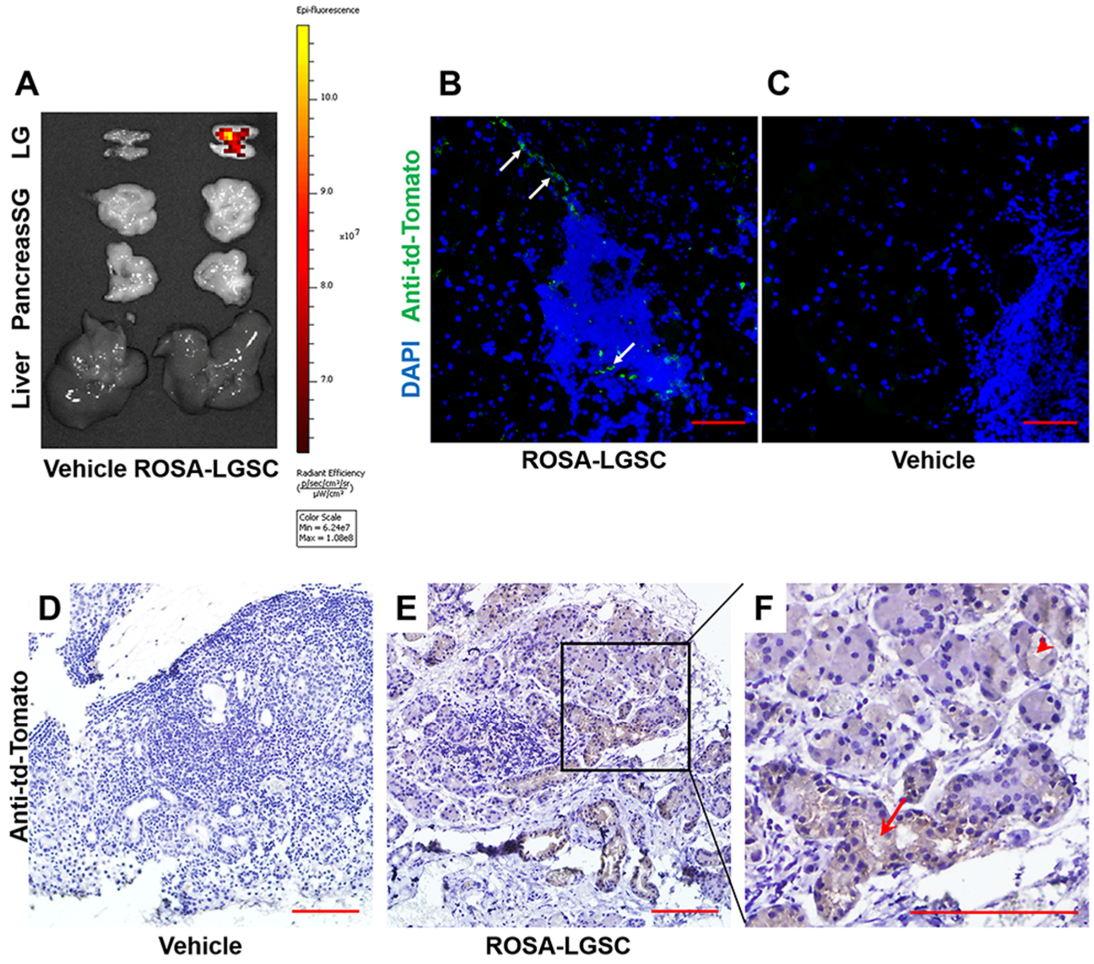

Figure S4. Engraftment of LGSC allotransplantation. A. The fluorescent images of NOD/ShiLtJ organs at 10 days post tail vein injection of ROSA-LGSCs. LG, lacrimal gland; SG, salivary gland. B, C. Immunofluorescent staining with anti-td-Tomato antibody of NOD/ShiLtJ LG tail vein injected with ROSA-LGSCs cultured for 7 days after 10 days. B. LG injected with ROSA-LGSCs (white arrow, green, exogenous cells). C. LG injected with vehicle; scale bar, 100 µm. D–F. IHC staining with anti-td-Tomato antibody of NOD/ShiLtJ LG transplanted with ROSA-LGSCs cultured for 7 days after 8 weeks. D. LG injected with vehicle. E. LG injected with ROSA-LGSCs. F. the magnified image of the black frame in E (red arrow, intra-lobular duct; red arrowhead, acini); scale bar, 100 µm
