# Supplementary material for: Establishment of long-term serum-free culture for lacrimal gland stem cells aiming at lacrimal gland repair
Source: Stem Cell Res Ther. 2020 Jan 8;11:20. doi: 10.1186/s13287-019-1541-1 (PMC6951017; doi:10.1186/s13287-019-1541-1)
Supplement: Supplementary file 9 — Figure S6. Isolation and characterization of NOD-LGSGs. A, B. The morphology of LGSCs and NOD-LGSCs in primary culture at day 7; scale bar, 400 μm. A. NOD-LGSCs; B. LGSCs. C. The sphere number per-field of NOD-LGSCs is significantly less than LGSCs. Scale bar, 400 μm; ***, P < 0.01; n = 5. D. Transcriptional expression of adult stem/progenitor cell markers of LGSCs and NOD-LGSCs. NC, negative control. E. Transcriptional expression of adult stem cell and differentiated markers of NOD-LGSCs cultured for 5, 7, 10, and 14 days. Secretory cell marker AQP5 and secretory protein gene of LG Ltf are significantly up-regulated. Adult stem cell marker Krt14 is significantly down-regulated. There is no significant change of ductal cell marker Krt19; ***, P < 0.01; ns, non-significance; n = 3. F. Immunofluorescent staining of NOD-LGCSs cultured for 5, 7, 10, and 14 days. As elongating culture time, cells expressing Krt14 (red) and Ki67 (red) are significantly decreased, and no cells expressing Krt19 (red) emerge, scale bar, 50 μm. Nuclear staining, DAPI (blue). G. The fluorescent images of NOD-LGSCs cultured for 7 days after being labeled with mCherry. BF, bright field (PDF 9808 kb) [file 13287_2019_1541_MOESM9_ESM.pdf]

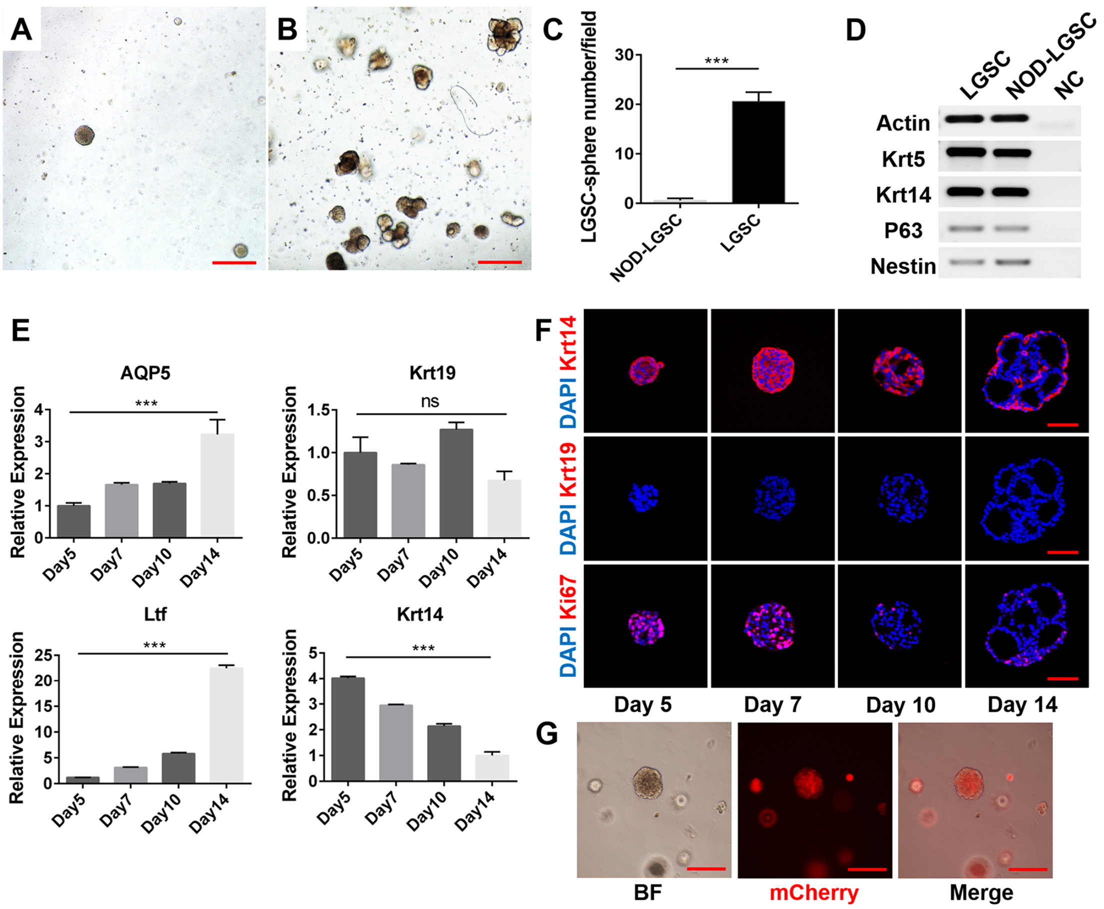

Figure S6. Isolation and characterization of NOD-LGSGs. A, B. The morphology of LGSCs and NOD-LGSCs in primary culture at day 7; scale bar, 400 μm. A. NOD-LGSCs; B. LGSCs. C. The sphere number per-field of NOD-LGSCs is significantly less than LGSCs. Scale bar, 400 μm; ***, $P < 0.01$; $n = 5$. D. Transcriptional expression of adult stem/progenitor cell markers of LGSCs and NOD-LGSCs. NC, negative control. E. Transcriptional expression of adult stem cell and differentiated markers of NOD-LGSCs cultured for 5, 7, 10, and 14 days. Secretory cell marker AQP5 and secretory protein gene of LG Ltf are significantly up-regulated. Adult stem cell marker Krt14 is significantly down-regulated. There is no significant change of ductal cell marker Krt19; ***, $P < 0.01$; ns, non-significance; $n = 3$. F. Immunofluorescent staining of NOD-LGCSs cultured for 5, 7, 10, and 14 days. As elongating culture time, cells expressing Krt14 (red) and Ki67 (red) are significantly decreased, and no cells expressing Krt19 (red) emerge, scale bar, 50 μm. Nuclear staining, DAPI (blue). G. The fluorescent images of NOD-LGSCs cultured for 7 days after being labeled with mCherry. BF, bright field.
